# American Medical Association

**Published:** 1860-05

**Authors:** 


### EDITORIAL AND MISCELLANEOUS.

### American Medical Association.

The American Medical Association will hold its thirteenth annual meeting at New Haven, Connecticut, on the first Tuesday of June, 1860. The secretaries of local societies, colleges and hospitals, are requested to forward the names of delegates as soon as they are appointed, to Stephen G. Hubbard, M. D., Secretary, New Haven, Connecticut.

The approaching meeting of this body, will probably be the most important which has been held for many years.—At this session the vexed question of Medical Education will in all probability, be disposed of, either by the assumption of the power upon the part of the association to control the whole subject, and the attempt to establish a uniform system for all the colleges in the United States, and the consequent destruction of the body itself, or, the association may act more judiciously, determine to ignore the whole question, confine itself for the future, to the advancement of science and to its legitimate sphere, and thus possibly perpetuate its existence.

